# Polar vs Non-polar Excipients in BSA Aggregation

**DOI:** 10.1007/s11095-026-04204-2

**Published:** 2026-10-07

**Authors:** Samuel Tien, Simone Nørgaard Krabbe, Annika Sollann Sørensen, Thomas Balle, Veysel Kayser

**Affiliations:** 1 Faculty of Medicine and Health, The University of Sydney, Sydney School of Pharmacy, Sydney, NSW 2006 Australia; 2 Present Address: Faculty of Health and Medical Sciences, The University of Copenhagen, Department of Pharmacy, 2100 Copenhagen, Denmark

**Keywords:** excipient-protein interactions, formulation, protein aggregation, simulation

## Abstract

**Purpose:**

In recent years, there has been a rapid expansion in the development of new protein therapeutics in the pharmaceutical industry. Problematically, proteins are conformationally and colloidally marginally stable, and thus it is necessary to formulate them using buffering and stabilizing agents.

**Methods:**

In this study, we use experimental and computation techniques to evaluate the aggregation pathway, as well as the stabilizing properties of a range of excipients on a model protein; bovine serum albumin (BSA).

**Results:**

Our results show that polar excipients (L-arginine and trimethylamine N-oxide) decrease BSA stability, whereas non-polar excipients (L-alanine and L-valine) enhances it. To elucidate molecular mechanisms behind these trends, we constructed a multi-step unfolding and aggregation model. Upon thermal stress, the protein system is driven to form a disrupted dimer/trimer species across a steep activation energy barrier. This acts as the primary kinetic bottleneck, as once these intermediate states form sufficiently, large aggregates in tetramers and pentamers form across a significantly lower energy barrier. High temperature molecular dynamics simulations reveal that the unfolding mode of the monomer is characterized by the detachment of its C-terminus domain, creating an extended, aggregation prone structure. Crucially, in the native structure, this flexible region contains the only prominent solvent exposed hydrophobic patch on an otherwise hydrophilic protein surface. Therefore, hydrophobic excipients are predicted to interface with this hotspot, stabilizing the folded structure and suppressing downstream aggregation formation.

**Conclusions:**

Overall, this work demonstrates that understanding the specific protein aggregation mechanisms is key for the rational selection of stabilizers.

**Supplementary Information:**

The online version contains supplementary material available at https://doi.org/10.1007/s11095-026-04204-2.

## Introduction

In the past couple of decades, protein therapeutics have given rise to a new way of treating a myriad of medical conditions due to their tunability and target-specific mode of action. The function of these therapeutics inherently relies on the native folded structure of the protein reaching the biological target [1, 2]. However, a central challenge in the development and manufacturing process is protein aggregation, which is the self-association of monomers into larger species, compromising its efficacy and safety. Aggregation may occur in both folded and unfolded states of the protein, though it typically arises from partial unfolding, exposing hydrophobic patches on the surface, which facilitates greater protein–protein interactions [3–5].

Protein stabilization strategies generally rely on two approaches: engineering of the protein structure, or modifying the external environment. Structural features of the protein, especially hydrophobic surfaces, affect the tendency of aggregation. Therefore, modifications of the amino acid sequence or the introduction of other stabilizing motifs, like through glycosylation, can improve conformational and colloidal stability [6, 7].

The other strategy involves formulating the therapeutics using buffering and stabilizing agents. However, the ideal choice of these excipients, as well as their effective working concentrations, is highly protein-dependent [8, 9]. Consequently, predictive aggregation models have been increasingly developed to decipher these protein-excipient interactions [10, 11].

In this study, we investigate the protein-stabilizing abilities of various excipients on the globular protein bovine serum albumin (BSA). BSA was chosen as a model protein due to its well-characterized structure, comprising of a single chain with 583 amino acid residues, and a molecular weight of 66.43 kDa. Additionally, BSA contains two tryptophan residues which act as fluorophores: Trp134 located on the surface, and Trp213 buried within the hydrophobic core of the protein.

**Fig. 1:**
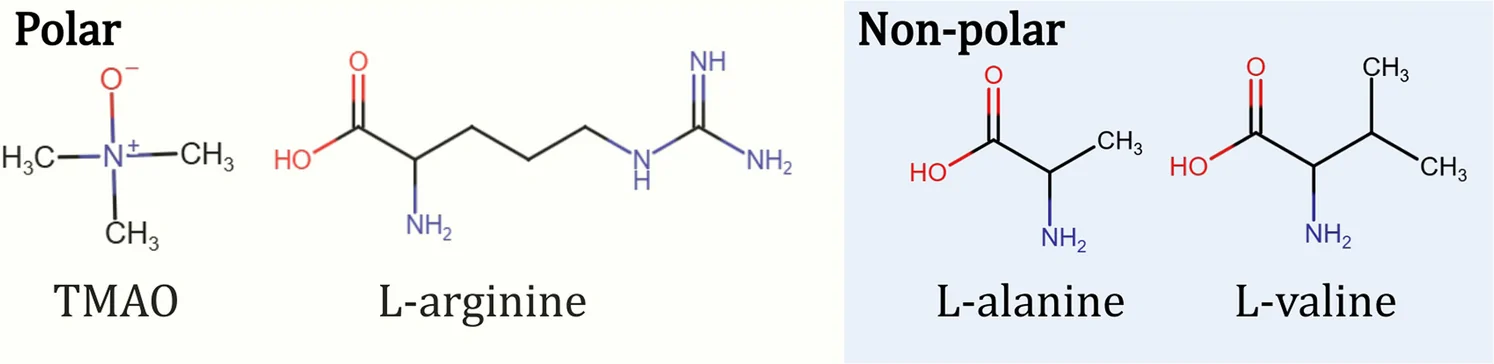
Chemical structures of the evaluated excipients: TMAO, L-arginine (polar compounds), and L-alanine, L-valine (non-polar compounds).

The excipients selected for this study comprises the prospective osmolyte stabilizer trimethylamine N-oxide (TMAO), a polar amino acid (L-arginine), and two aliphatic non-polar amino acids (L-alanine and L-valine) (Fig. 1).

L-arginine is an established stabilizer used in many protein formulations [12]. It is highly positively charged at physiological pH with the zwitterionic amino acid backbone at one end, and the protonated guanidinium side chain at the other. TMAO is a small amphiphilic molecule, which consists of a highly charged amine oxide group and a bulky hydrophobic trimethyl group. TMAO has been shown to stabilize a range of protein systems [13–15].

On the other hand, non-polar excipients L-alanine and L-valine represent an unconventional class of stabilizers for liquid formulations, being commonly used with lyophilized proteins [16]. Structurally, L-valine possesses a higher hydrophobicity than L-alanine due to its branched isopropyl side chain, though both have relatively low solubility compared to polar molecules such as TMAO. While these are classified as non-polar, they retain a zwitterionic amino acid backbone at physiological pH, as to be somewhat miscible with water.

In this study, we pair empirical stability observations with a complementary set of experimental and computational analyses to deduce a multi-step aggregation model for BSA. By doing so, we establish how these excipient’s charged nature rationally dictates protein stability.

## Results

### The Effect of Excipients on BSA Stability

The melting temperatures (T_m_) of BSA in the presence of the excipients were determined by a temperature ramp, monitoring the intrinsic fluorescence intensity ratio I_350_/I_330_. The inflection point of this transition is indicative of unfolding or aggregation, as tryptophan emission is highly sensitive to the polarity of its surrounding environment [17].

**Fig. 2:**
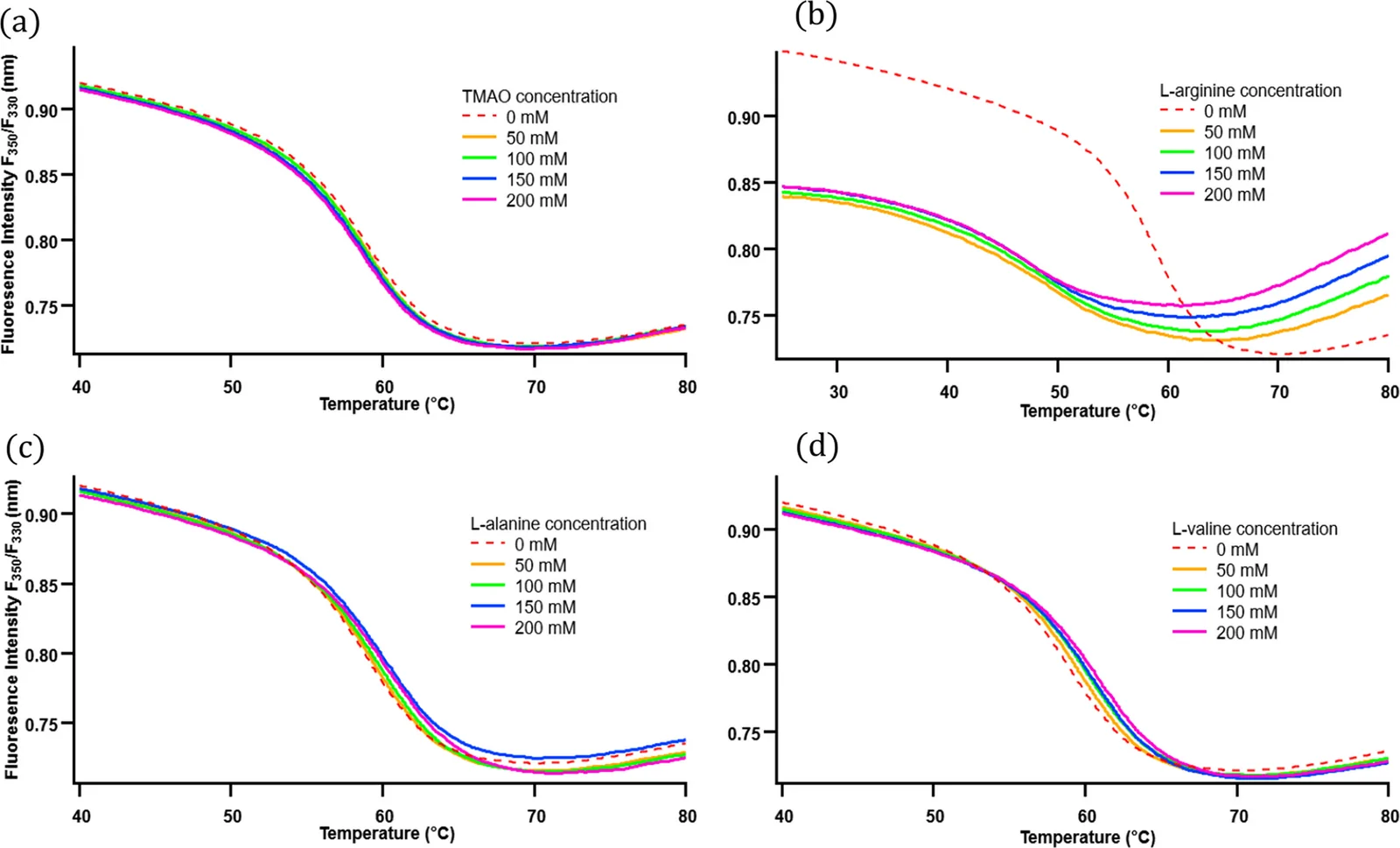
Thermal degradation profiles of BSA across varying excipient concentrations. Temperature ramps were performed at 0, 50, 100, 150, and 200 mM of (**a**) TMAO, (**b**) L-arginine, (**c**) L-alanine, and (**d**) L-valine. Intrinsic fluorescence intensity ratios (F_350_/F_330_) were smoothed using a 2nd-order Savitzky–Golay filter with a window width of 31 points. Data are displayed from 40 °C to 80 °C for all panels, except for L-arginine, which is displayed from 25 °C to 80°C. The raw data is shown in Supplementary Fig. 1.

Native BSA displayed an initial fluorescence ratio of 0.95, which subsequently dropped to 0.72 upon thermal degradation (Fig. 2). This is reflected in all other profiles except for L-arginine, where across all concentrations, the initial baseline was 0.84 (Fig. 2b). This downwards shift suggests that L-arginine either directly quenches tryptophan residues or induces unfolding/aggregation of the native protein prior to thermal stress.

Interestingly, a downward trend in the fluorescence intensity ratio with increasing temperature is atypical for standard protein denaturation. Classical unfolding models predict that thermal stress drives internal tryptophan residues to the aqueous environment, leading to fluorescence quenching and a characteristic spectral red shift. The observed downward ratio trend indicates an alternative process, driven either by a spectral blue shift, selective fluorescence quenching near 350 nm, or aggregation induced light scattering artifacts.

From the fluorescence inflection point, the melting temperature of native BSA was determined to be 58.9°C (Table 1). For polar excipients TMAO and L-arginine, the inflection point is shifted towards a lower melting temperature, indicating a destabilizing effect. TMAO induced small changes in the melting temperature, decreasing to 58.7°C at 50 mM and 58.4°C at 200 mM. In contrast, L-arginine lowers the melting temperature drastically, decreasing to 49.2°C at 50 mM and 46.9°C at 200 mM. In general, both polar excipients followed a concentration-dependent trend where elevated concentrations correlated with a decrease in thermal stability.

**Table I:** Melting temperatures of BSA in excipients extracted from the intrinsic fluorescence intensity ratio (F_350_/F_330_)

*Prometheus Panta*	Melting temperature (°C)			
Control	58.9			
*Excipient concentration (mM)*	TMAO	L-arginine	L-alanine	L-valine
*50*	58.7	49.2	59.3	59.6
*100*	58.6	48.7	59.6	60.1
*150*	58.5	47.8	59.8	60.3
*200*	58.4	46.9	60.0	60.7

For non-polar excipients L-alanine and L-valine, the melting temperature increased with excipient concentration, shifting the inflection point towards the right, indicating its stabilizing effect. For L-alanine, the melting temperature increases to 59.3°C at 50 mM and 60.0°C at 200 mM, while L-valine was the most effective stabilizer, increasing melting temperature to 59.6°C at 50 mM and 60.7°C at 200 mM. The general ranking of stabilization follows: L-arginine < TMAO < control < L-alanine < L-valine, matching the polarity of the excipients.

To confirm this effect, ANS (8-Anilino-1-naphthalenesulfonic acid) and ThT (Thioflavin T) fluorescence assays were employed to monitor conformational unfolding and aggregation respectively, during thermal stress in the presence of 200 mM excipients. ANS is a dye which is practically non-fluorescent in solution, however upon binding to hydrophobic patches on proteins, the mobility of the molecule becomes restricted, which causes an increase in fluorescence intensity [18, 19]. ThT operates on similar principles but specifically binds to β-sheet rich regions formed during aggregation.

**Fig. 3:**
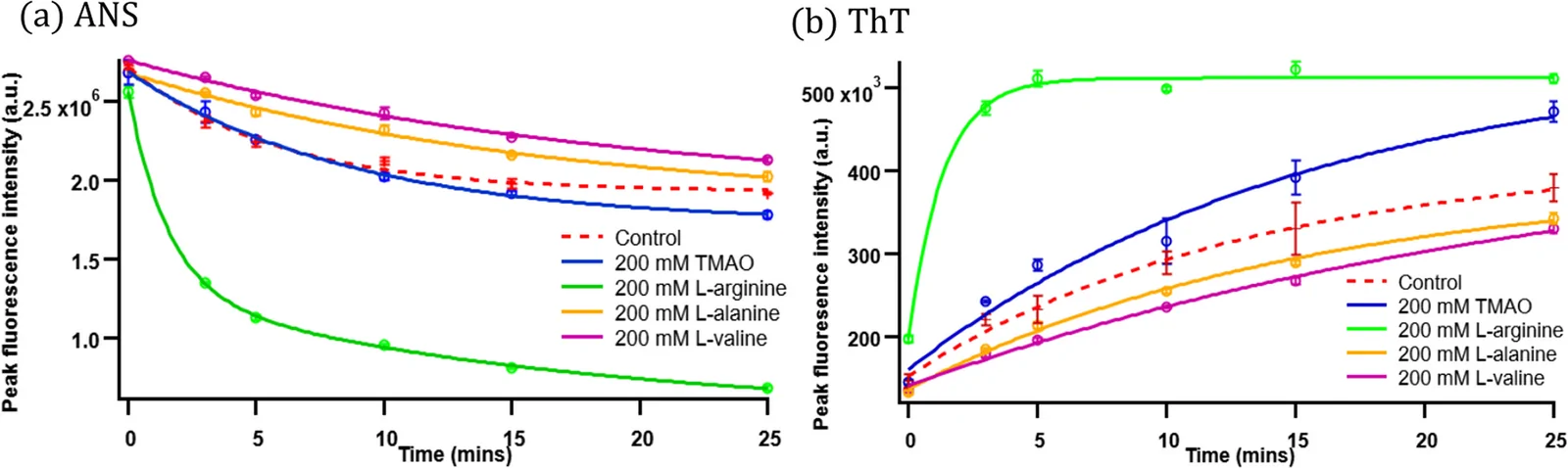
Unfolding and aggregation kinetics of BSA with 200 mM of TMAO, L-arginine, L-alanine and L-valine. Peak fluorescence intensities over incubation time are shown for the extrinsic fluorophores (**a**) ANS using an incubation temperature of 60 °C and (**b**) ThT using an incubation temperature of 63°C. Error bars show the standard deviation from triplicate measurements.

Interestingly, ANS peak fluorescence decreases over time during thermal incubation (Fig. 3a), in which formulations with L-arginine yielded the largest decrease in intensity, followed by TMAO. Conversely, L-alanine and L-valine peak fluorescence intensities lie above the control, with L-valine showing the smallest decrease in intensity over time, consistent with the melting temperature findings.

ThT peak fluorescence increased with incubation time (Fig. 3b). Consistent with the previous data, L-arginine and TMAO accelerated aggregation, increasing peak fluorescence above the control, while L-alanine and L-valine decreased peak fluorescence, suggesting that the resistance to unfolding directly translates into reduced aggregation propensity.

Additionally, the baseline ANS and ThT fluorescence of L-arginine lie above and below the other excipients respectively, further supporting that L-arginine disrupts the native state of BSA.

### Aggregation Mechanisms of BSA

#### Intrinsic Fluorescence Spectra of Aggregation

As nanoDSF on Prometheus Panta uses fixed wavelength measurements at 350 nm and 330 nm, to assess the complete intrinsic tryptophan fluorescence profile of aggregation, an additional thermal ramp was conducted for BSA, collecting the complete fluorescent spectra from 25–97°C in 3 °C intervals.

**Fig. 4:**
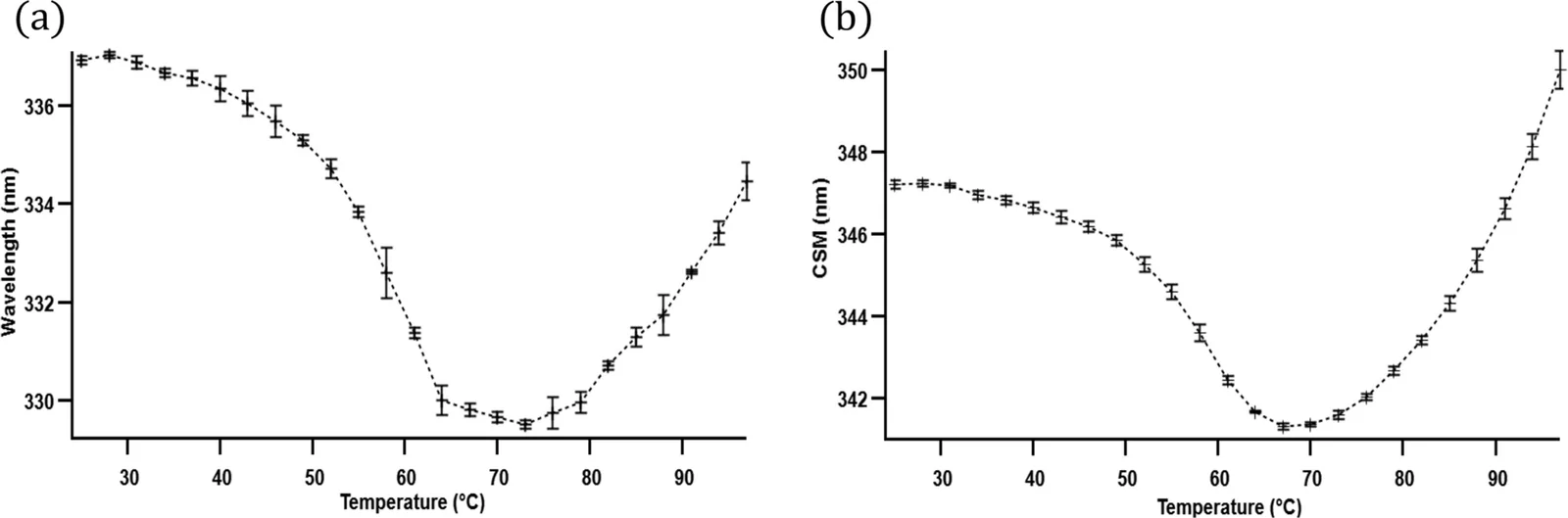
Intrinsic tryptophan fluorescence profiles of BSA from a temperature ramp of 25–97°C, showing (**a**) peak wavelength over temperature, (**b**) center of spectral mass (CSM) over temperature. Error bars indicate the standard deviation from triplicate measurements. The raw spectrum for all temperatures is shown in Supplementary Fig. 2.

Both the peak emission wavelength (${\lambda}_{max}$) and the center of spectral mass (CSM) demonstrate a biphasic trend (Fig. 4). The spectra initially blue shifts from 337 to 330 nm in temperatures of 25–76°C in a sigmoid-like function, before undergoing a red shift at the tail end of the transition. The initial sigmoidal transition confirms that the decrease in the fluorescence intensity ratio is driven by the blue shift of the emission spectra. The late-stage red shift likely corresponds to the formation of large insoluble aggregates or late-stage denaturation of aggregates.

#### Arrhenius Mechanics in ANS and ThT Fluorescence

To further understand why non-polar excipients are more effective in the stabilization of BSA, we seek to understand its aggregation mechanisms. One of the most common frameworks to predict unfolding and aggregation profiles of proteins is through Arrhenius kinetics, which relates temperature to reaction rate through an activation energy:

1 $$k=A\cdot \mathrm{exp}\left(-\frac{{E}_{a}}{RT}\right)$$

where *k* is the reaction rate, *A* is the pre-exponential factor, ${E}_{a}$ is the activation energy, *R* is the universal gas constant, and *T* is the temperature.

Importantly, this assumes a one step process, with one start and end state for the reaction pathway (*A ↔ B*). This is widely used to extrapolate reaction kinetics to higher or lower temperatures.

Subsequently, time dependent ANS and ThT dye binding assays were conducted across a range of temperatures (55–65°C for ANS, 58–70°C for ThT). A concentration of 0.2 mg/mL BSA was used for ANS, and a concentration of 2 mg/mL BSA was used for ThT. The kinetics were modelled with a single exponential decay function, which provided the best fit for the given data.

**Fig. 5:**
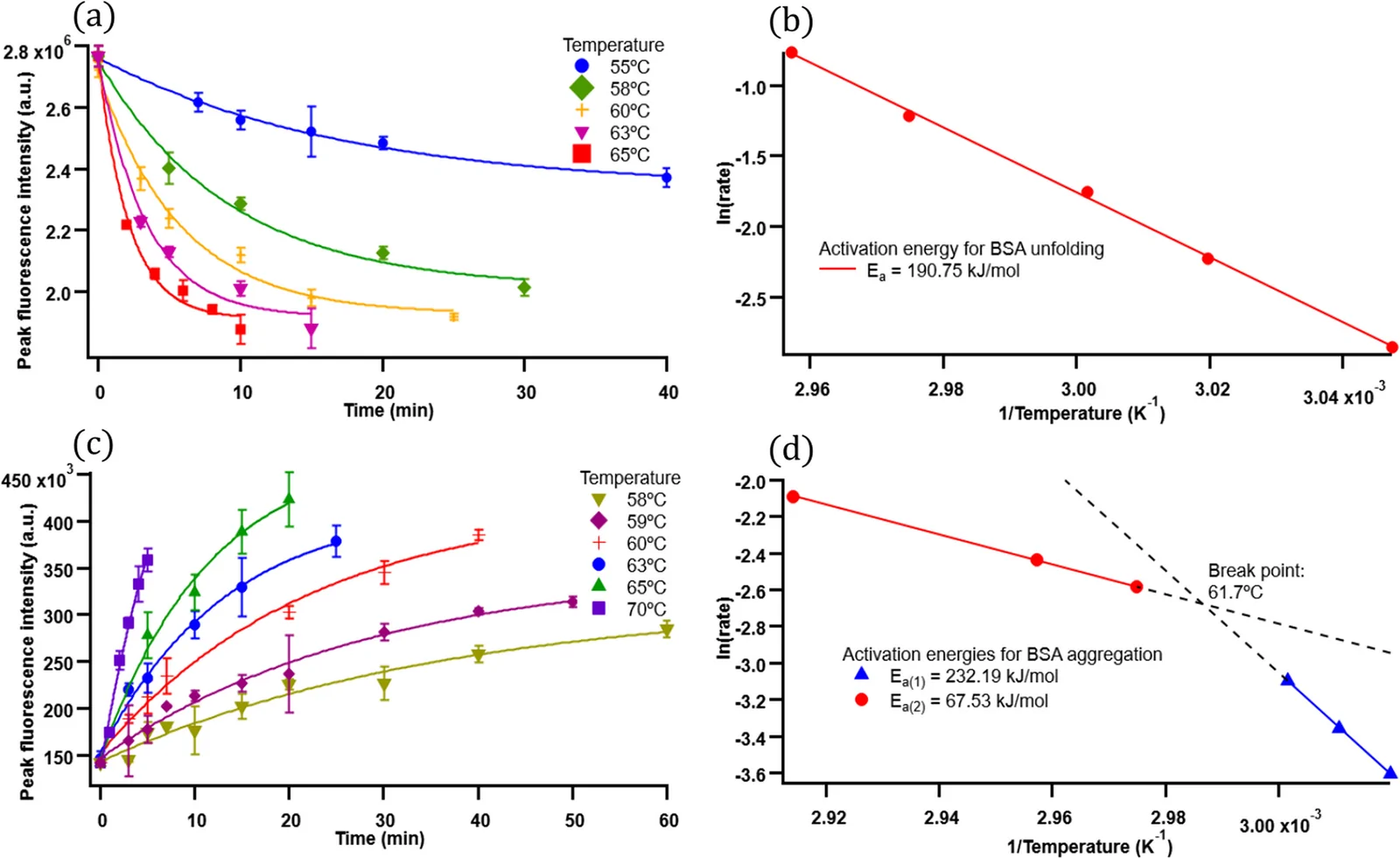
Kinetics of BSA unfolding and aggregation. (**a**) ANS peak intensity over time for temperatures of 55, 58, 60, 63, and 65°C. (**b**) Arrhenius plot derived from ANS rates, showing linear behavior and an activation energy of 190.75 kJ/mol. (**c**) ThT peak intensity over time for temperatures of 58, 59, 60, 63, 65, and 70°C. (**d**) Arrhenius plot derived from ThT rates showing 2 linear fits with a kinetic break point at 61.7°C. Calculated activation energies are 232.19 kJ/mol below, and 67.53 kJ/mol above this point. Error bars show standard deviations from triplicate measurements.

As seen previously, ANS fluorescence intensity decreased as a function of increased temperature and time (Fig. 5a). The resulting Arrhenius plot (Fig. 5b) shows a linear trend within the temperature range, indicating that BSA unfolding adheres to Arrhenius kinetics. From the slope, the activation energy for unfolding was determined to be 190.75 kJ/mol.

ThT fluorescence intensity increased as a function of both temperature and time (Fig. 5c). The Arrhenius plot (Fig. 5d) displays two separate linear regimes with different slopes and activation energies. From extrapolating these two lines, there is a break point identified at 61.7°C, where at temperatures below this point the activation energy is 232.19 kJ/mol, and above the activation energy is 67.53 kJ/mol. This indicates a lower kinetic barrier to aggregation at higher temperatures. Notably, this break point occurs above the melting temperature of 58.9°C. While treated here as a discrete intersection of two Arrhenius lines, this break point likely represents a simplified model of a continuous transition between competing rate limiting steps in the pathway.

This suggests the aggregation mechanism does not follow Arrhenius kinetics directly and is most likely a multi-step process. In the literature, Arrhenius graphs with a clear intersection point can either be modelled with a double activation energy as implemented here, or using a hyperbolic function [20–22]. We did not implement the latter, as fitting a mathematical function over an incomplete model will often lead to overfitting.

#### SEC-MALS

To investigate the non-Arrhenius kinetics of aggregation, size-exclusion chromatography coupled with multi-angle light scattering (SEC-MALS) was used to monitor the colloidal stability of BSA. This allows for the extraction of the molecular weight and populations of each aggregate species. Instrument and method precision was verified using triplicate injections of the native BSA reference, yielding a reproduceable molecular weight (6.69 ± 0.03 ⋅ 10^4^ g/mol, RSD < 1.5%), a close estimate of the theoretical molecular weight. Molecular weights were determined in Zimm plots analysis (Supplementary Fig. 3).

**Fig. 6:**
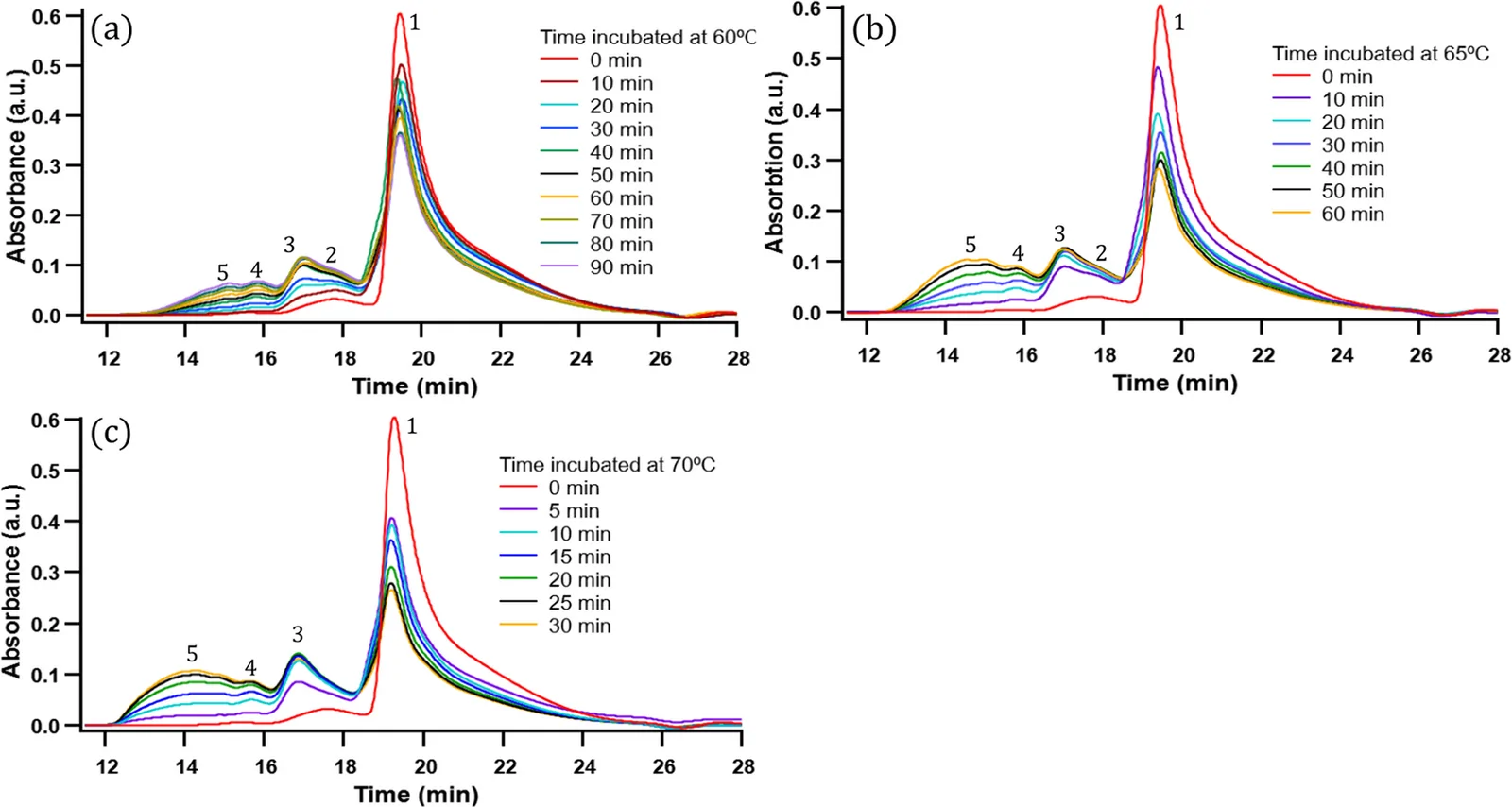
Representative SEC-HPLC UV chromatograms of BSA aggregation. Elution times are shown between 11.5–28 min for BSA samples incubated at (**a**) 60 °C (0–90 min), (**b**) 65 °C (0–60 min), and (**c**) 70 °C (0–30 min). Labelled peaks correspond to the molecular weight assignments characterized in Table 2, and the kinetic profile of each species is given in Fig. 7.

Subsequently, BSA samples were incubated for various times at 60 °C (below the kinetic break point), 65 °C, and 70 °C (above the kinetic break point). Unstressed, native BSA exhibits a dominant monomeric peak (Fig. 6, Peak 1) alongside a minor population of a higher-order native state (Peak 2). Upon thermal incubation at 60 °C, three additional peaks emerge at earlier elution times (Peaks 3–5), indicating the formation of larger aggregate species. Notably, Peak 2 becomes progressively less pronounced at 65 °C and becomes completely indistinguishable at 70 °C, where it co-elutes with Peak 3, and was integrated as a single combined population.

The molecular weights for each aggregated species were determined with multi-angle light scattering in Zimm plot analysis (Supplementary Fig. 4) and summarized in Table 2. Peak 1 corresponds to the monomer species validated previously. The oligomeric stoichiometry of the other peaks was determined by dividing by this value.

**Table II:** Molecular weights assignments and oligomeric states for each BSA species derived from MALS. The number of subunits corresponds to the peaks identified in the SEC-HPLC UV chromatograms. Corresponding Zimm plots for each aggregated species are provided in Supplementary Fig. 4

*Peak number*	Elution time (mins)	Molecular weight (g/mol)	Number of subunits
*1*	19	6.69 ⋅ 10^4^	1
*2*	18	1.68 ⋅ 10^5^	2.5
*3*	17	1.62 ⋅ 10^5^	2.4
*4*	16	2.60 ⋅ 10^5^	3.9
*5*	15	3.32 ⋅ 10^5^	5.0

Interestingly, Peaks 2 and 3 exhibit highly similar molecular weights, corresponding to a dimer/trimer species. This is a known phenomenon in proteins, where different oligomeric states may display distinct substructures [23]. As they have different elution times, this indicates that two separate structural states are forming: a native, and a disordered counterpart.

Given that Peak 2 is present in the unstressed control and the equilibrium shifts toward Peak 3 at elevated temperatures, Peak 3 likely represents the disordered aggregate population. Higher order aggregates, Peaks 4 and 5, possess molecular weights consistent with 4 and 5 subunits respectively, identifying them as tetramers and pentamers.

Furthermore, to determine the structural properties of distinct aggregate populations, oligomeric species were isolated with fraction collection, spanning the monomeric (19–20 min), dimer/trimer (17–18 min), and tetramer/pentamer (13–16 min) elution windows for a sample incubated at 70°C. Analysis of their respective intrinsic tryptophan fluorescence spectra (Supplementary Fig. 5) reveals that the monomeric and dimer/trimer fractions shares an equivalent peak wavelength at ~ 337 nm. This indicates that dimerization and trimerization does not significantly alter the local environment of the tryptophan residues.

In contrast, the higher-order tetramer/pentamer fraction displays a pronounced blue shift to ~ 330 nm. This suggests that the movement of tryptophan into non-polar microenvironments occurs specifically during assembly into these higher-order species.

**Fig. 7:**
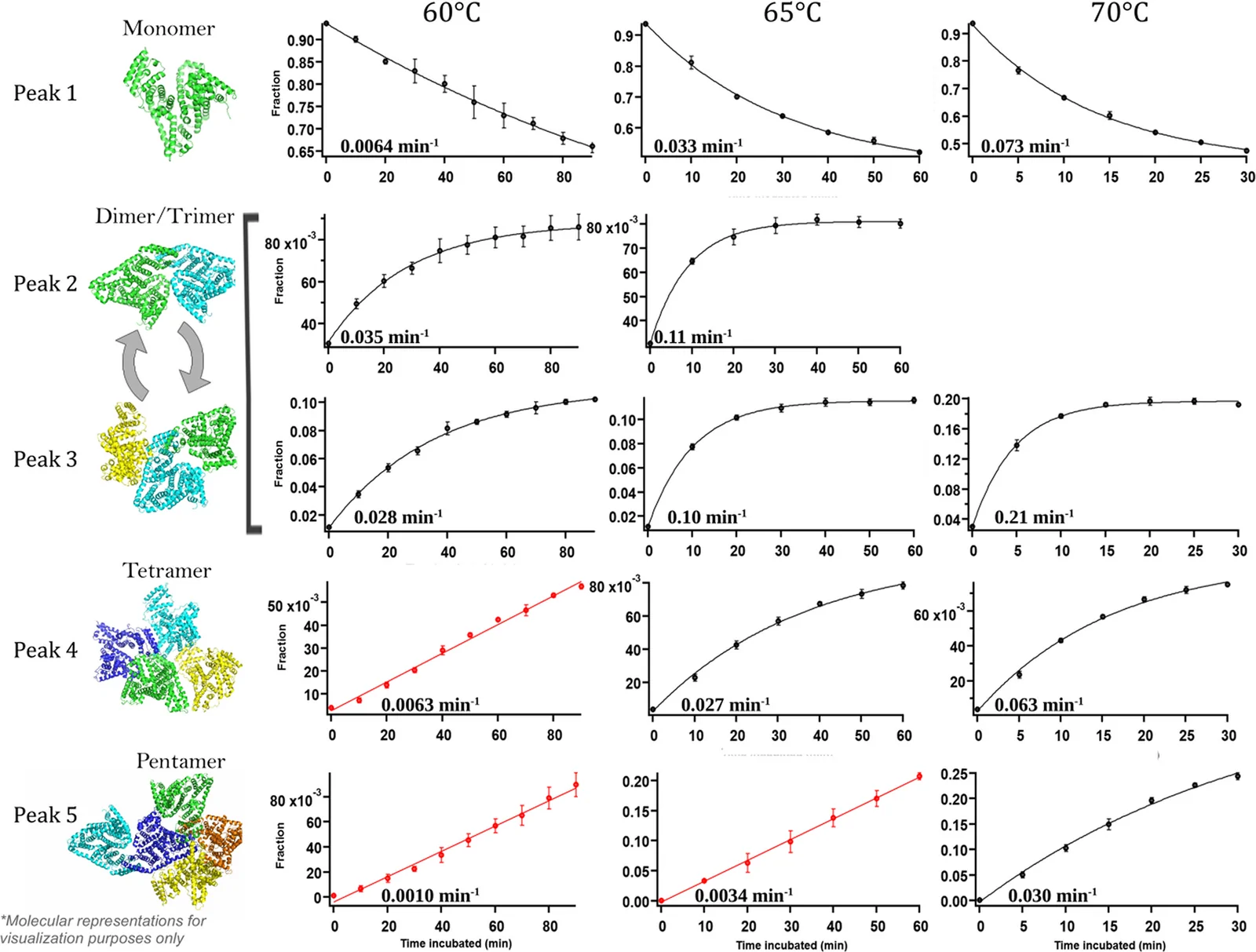
Kinetic evolution of BSA populations for each species over time, showing incubation at 60 °C (0–90 min), 65 °C (0–60 min), and 70 °C (0–30 min). Exponential fits are shown in black, and linear fits are given in red. Calculated rate constants are shown in the graphs. Error bars show the standard deviation from triplicate measurements.

The kinetics of aggregate formation were assessed by plotting the population against time (Fig. 7). Populations were determined from integrating the peak areas from the UV chromatogram. Depending on the kinetic profile, rates were extracted with either a single exponential decay function or a linear fit.

As expected, the monomer populations decay with prolonged incubation time and temperature, where the calculated rates were 0.0064 min^−1^ at 60ºC, 0.033 min^−1^ at 65ºC, and 0.073 min^−1^ at 70ºC (Fig. 6). Subsequently, small aggregate formation (Peaks 2 and 3) corresponds to monomer loss, with populations accumulating as a function of incubation time. The extracted kinetic rates for these species increase proportionally with elevated temperatures.

Interestingly, the formation of tetramers (Peak 4) is linear at 60ºC, with a rate of 0.0063 min^−1^, while it is exponential at 65ºC and 70ºC, with a rate of 0.027 min^−1^ and 0.063 min^−1^ respectively. Likewise, the formation of pentamers (Peak 5) is linear at 60ºC and 65ºC, with a rate of 0.0010 min^−1^ and 0.0034 min^−1^, respectively, and exponential for samples incubated at 70ºC, with a rate of 0.030 min^−1^. The linear function of population increase at lower temperatures suggests it is only capturing the initial phase of the pseudo exponential decay, meaning these aggregation pathways are occurring slowly and have not yet reached completion.

#### Unfolding Simulations

To characterize the specific aggregation mode of BSA, accelerated unfolding simulations were performed to generate conformations of the unfolded monomeric state. Four independent, short MD simulations were initiated from the crystal structure at an elevated temperature of 500 K. Trajectory analyses (Supplementary Fig. 6) confirmed the progression of thermal denaturation, as evidenced by a time-dependent increase in root-mean-square deviation (RMSD). Concurrently, the radius of gyration (Rg) increased from an initial native value of 2.6 nm to a range between 2.7 and 3.2 nm.

**Fig. 8:**
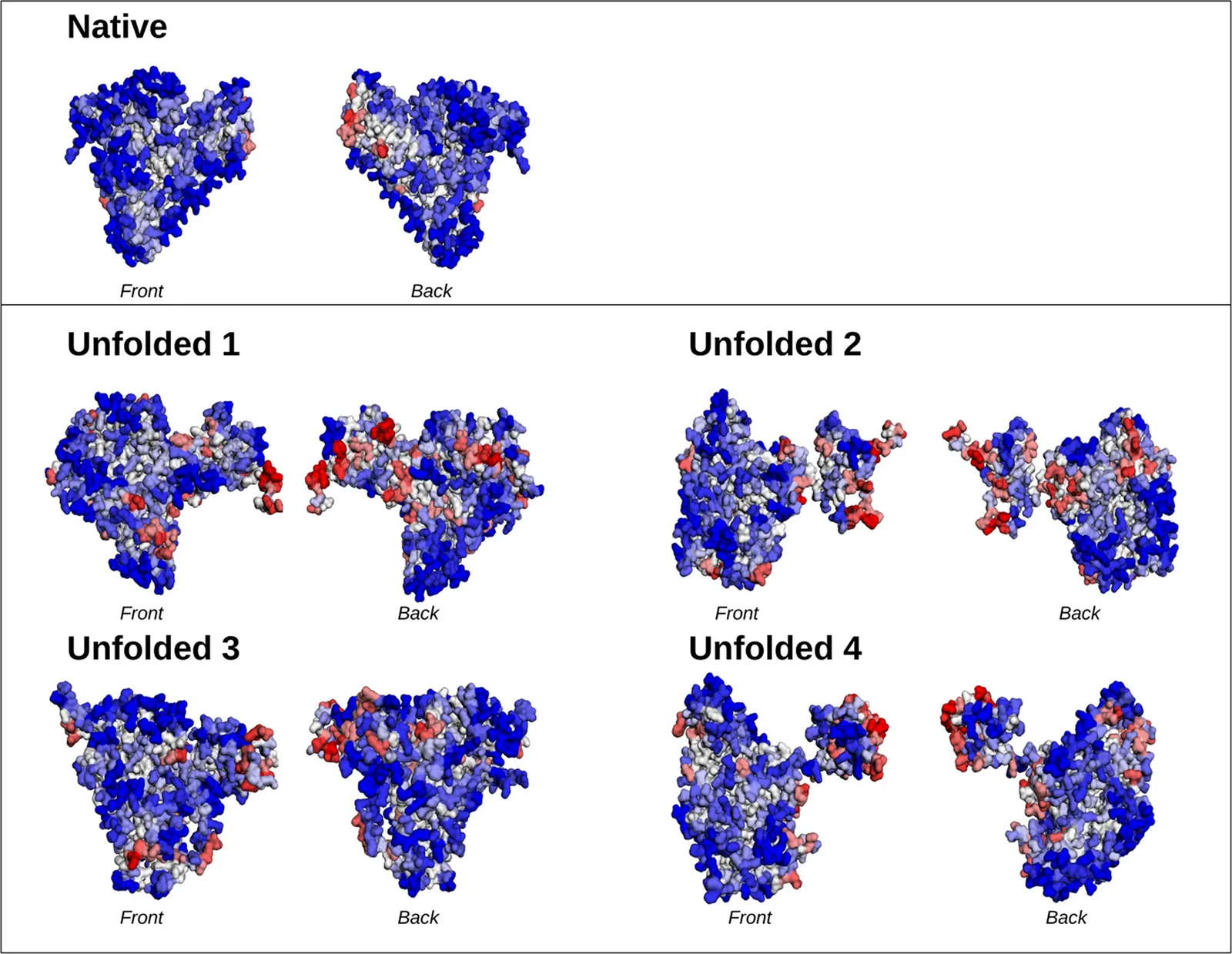
Aggrescan3D scores projected onto the protein surface in native and unfolded structures. Blue residues represent negative scores (low aggregation propensity), red residues represent positive scores (high aggregation propensity), and white residues represent scores of 0 (not solvent exposed).

The resulting structures are displayed in Fig. 8 and were characterized using Aggrescan3D [24] and FreeSASA [25], with the global structural metrics summarized in Supplementary Table 1. Aggrescan3D is a structure-based algorithm that evaluates individual residues based on their local aggregation propensity by identifying solvent-accessible hydrophobic patches, which are denoted by positive scores.

Analysis of the native BSA structure revealed that the majority of the solvent-accessible surface area (SASA) is predominantly hydrophilic and thus resistant to self-association. However, a distinct hydrophobic solvent accessible hotspot was identified in proximity to the C-terminus, comprising residues Val569, Val575, Val576, Ala581, and Ala583 (Supplementary Fig. 7).

Upon unfolding, a consistent structural transition was observed across all simulations (Fig. 8). Specifically, the C-terminal domain (Thr491-Ala583) detaches from the main protein body, directly accounting for the observed expansion in the radius of gyration. Aggrescan3D analysis reveals that this detachment drives an overall increase in aggregation propensity scores in this area. However, this is not limited within the C-terminal domain itself but also across the adjacent Gln384-Thr491 region, where hydrophobic residues usually hidden within the protein core become solvent exposed, driving a further increase in aggregation propensity (Supplementary Fig. 8).

This unfolding movement explains why a distinct disrupted dimer/trimer species could be identified in SEC-MALS, with its extended structure. Most importantly, on the native state of the protein, this flexible unfolding region contains the only prominent hydrophobic solvent accessible region.

## Discussion

As addressed here, we will first establish an aggregation model of BSA, in which the differences in stabilizing agents can be explained.

An unusual feature of the thermal profile of BSA is the progressive decrease in peak wavelength and the I_350_/I_330_ fluorescence ratio of intrinsic tryptophan fluorescence. This is opposite to the typical red shift observed during protein denaturation where tryptophan residues are exposed to the aqueous environment upon unfolding. Structurally, this measurement probes the two intrinsic tryptophan residues: Trp134, which is relatively surface-exposed, and Trp213, located deep within the hydrophobic protein core. Our MD simulations show that the unfolding mode; detachment of the C-terminal region, does not increase the solvent exposure of Trp213, nor does Trp134 participate in this motion. Therefore, partial unfolding of the protein should not significantly alter the tryptophan microenvironment. Similarly, the initial dimer/trimer formation likely occurs from interfacing with the hydrophobic surfaces formed by the partially unfolded C-terminal. As before, both tryptophan residues are not structurally in proximity to this region and should not be involved in these conformational changes. This is supported by fluorescence analysis of the isolated fractions, which shows that the dimer/trimer species retains a peak tryptophan emission wavelength equivalent to the monomer.

In contrast, for isolated tetramers/pentamers, the intrinsic fluorescence wavelength displays a blue shift indicating a more hydrophobic microenvironment around tryptophan residues. This may reflect sequestration of the surface exposed Trp-134 into non-polar aggregate interfaces. Therefore, the melting temperatures measured by intrinsic fluorescence of BSA is in actuality an indicator of tetramer/pentamer formation, where partial unfolding and dimer/trimer formation do not appear to significantly alter the emission maximum.

ANS fluorescence intensity typically increases with incubation time, as the accessibility of hydrophobic surfaces increases during unfolding. However, counterintuitively, prolonged incubation time and elevated temperatures correspond to decreasing peak intensity. A plausible explanation suggests the thermal unfolding causes the collapse of internal hydrophobic binding pockets [26]. While thermal denaturation may introduce more binding spots on the surface overall, internal binding pockets will possess a higher quantum yield. It is unclear how many binding sites BSA has for ANS, where between 1 and 10 binding sites are reported, with six of them being with high affinity [27, 28]. This may explain the notably high ANS fluorescence for native BSA. Another explanation suggests that BSA adopts a compact structure to hide hydrophobic residues [29, 30], but according to the results of the unfolding simulations and the increased non-polar SASA, this is not likely the cause.

Regardless, the peak fluorescence of ANS adheres to a pseudo first order exponential decay function, in which the rates follow Arrhenius kinetics within the temperature range of 55–65ºC. Another study indicates that the unfolding of BSA follows Arrhenius kinetics with a break point at 67.3ºC [31], as such, our values are consistent with activation energies below this threshold.

Aggregation as probed by ThT fluorescence displays two Arrhenius regions, with a break point identified at 61.7ºC. The activation energy is drastically reduced above this temperature, in which values are consistent within previous studies, indicating aggregation proceeds more spontaneously [32]. Previously, this was explained by the rate limiting of unfolded species at lower temperatures (below 65ºC) [33].

However, with SEC-MALS, we can gain a more complete picture of the system, in which it is likely the temperature dependence in the formation of small aggregates (native and disrupted dimers/trimers), compared to larger aggregates (tetramers and pentamers). As from kinetic graphs (Fig. 7), at lower temperatures, the formation of low order aggregates (dimers/trimers) dominate. High order aggregates (tetramers and pentamers) increase linearly and are slow forming, meaning they are thermodynamically throttled. With higher temperatures, high order aggregates have exponential decay and are forming quickly. In addition, the native dimer/trimer peak gradually disappears. This may indicate that at low temperatures, the formation of larger aggregates is limited by an intermediate species, identified to be disrupted dimer/trimers. As they become more abundant at higher temperatures, the formation of larger aggregates is limited by collisions only. Therefore, disrupted dimer/trimer species act as the aggregation ‘seed’.

**Fig. 9:**
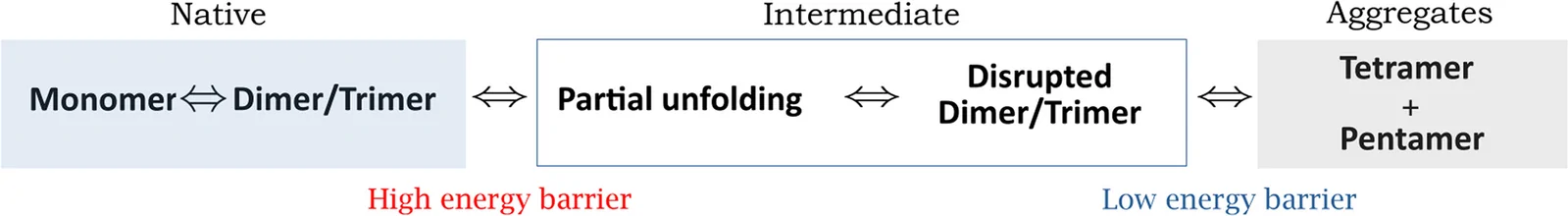
Proposed BSA aggregation model.

Building on this information, we can construct an aggregation model for BSA (Fig. 9).

In its native state, BSA is a combination of monomers and low population native dimers/trimers. As thermal stress is applied, the protein unfolds with an activation energy of 190.75 kJ/mol determined from ANS kinetics. Subsequently, within temperatures up to 61.7ºC, aggregation proceeds through predominately the formation of disrupted dimers/trimers, with steep activation energy of 232.19 kJ/mol. As the disrupted dimer/trimer species become more abundant, at temperatures surpassing the 61.7ºC break point, aggregation transitions towards the formation of tetramers and pentamers, which proceed across a significantly lower activation energy of 67.53 kJ/mol.

From this model, the BSA aggregation pathway is kinetically bottlenecked at the formation of disrupted dimers/trimers intermediates. High temperature MD simulations reveal the primary unfolding mode of BSA involves the detaching of the C-terminus domain, creating an extended, aggregation prone structure, a precursor to the disrupted dimer/trimer species. Crucially, while the solvent exposed surface of native BSA is predominantly hydrophilic, the mobile C-terminus domain is highly hydrophobic. This characteristic protein surface provides a clear rationale for the excipient screening results, where the efficacy of unfolding and aggregate suppression followed the order of increasing hydrophobicity: L-arginine < TMAO < control < L-alanine < L-valine.

While L-arginine is a common aggregation inhibitor used in protein formulations, our results confirm that it acts as a destabilizing denaturant in the specific case of BSA, aligning with previous studies [34–36]. Similarly, the prospective stabilizer TMAO marginally reduced BSA stability here. Although prior computational studies have suggested that TMAO exerts a protective effect on BSA [37, 38], they lacked validation via experimental thermal experiments. Charged excipients typically stabilize proteins by competing with water molecules at the protein surface, where they form clusters around charged residues [39, 40]. Moreover, they promote shorter and stronger hydrogen bonds between water molecules, essentially discouraging water molecules from outcompeting intra-protein hydrogen bonds [41]. However, in the case of BSA, a highly charged solvent shell will only increase the local polarity difference at the hydrophobic unfolding C-terminal domain, destabilizing the protein.

The superior performance of hydrophobic excipients on BSA specifically has been identified previously, in which stability increased with side chain hydrophobicity in the order Glycine < Alanine < Proline [42]. While hydrophobic excipients preferentially interact with non-polar sites [43], these amino acids still retain a zwitterionic backbone, which create a direct interface to water. Although not tested in the current study, we hypothesize that surfactants, which possess a more amphiphilic nature would offer even greater stabilizing capabilities.

This specific stabilization pattern is unique to BSA due to its unique unfolding and aggregation mechanisms. But overall, these findings demonstrate that a detailed mechanistic understanding of a protein’s aggregation mode must be considered in the rational selection of stabilizing excipients.

## Conclusion

In conclusion, we have demonstrated that hydrophobic excipients (L-alanine and L-valine) enhance the resistance of BSA to unfolding and aggregation, while hydrophilic excipients (L-arginine and TMAO) are detrimental under the conditions we used. Using experimental and computational techniques, we have elucidated the specific partial denaturation and aggregation pathway of BSA. The protein initially partially unfolds and forms a disrupted dimer/trimer species requiring a high activation energy of 232.19 kJ/mol. This intermediate state acts as a seed, where when they form sufficiently, larger aggregates in tetramers and pentamers are formed more spontaneously, requiring a lower activation energy of 67.53 kJ/mol. Molecular dynamics revealed that the initial unfolding event preceding the formation of these intermediate disrupted dimers/trimers is driven by the C-terminus domain (Thr491-Ala583) detaching from the main body of the protein, creating an extended structure with highly aggregation prone regions. Most importantly, in the native structure of BSA the protein surface is highly hydrophilic outside of this specific domain, and so hydrophobic excipients are predicted to interface with the unfolding hotspot. This interaction effectively shields this region, preventing further unfolding and the downstream formation of larger aggregates. Overall, this work shows that a fundamental understanding of protein-specific aggregation mechanisms is essential for the rational selection of excipients.

## Methods

### Materials

BSA lyophilized powder 96% (A2153), potassium phosphate dibasic ≥ 98.0% (P8281), potassium phosphate monobasic ≥ 98.0% (P9791), ThT (T3516), ANS ≥ 97% (A1028), trimethylamine N-oxide 95% (317,594), L-arginine ≥ 98% (A5006), L-alanine ≥ 99% (W381829), and L-valine ≥ 98% (V0500) were obtained from Sigma-Aldrich (Missouri, USA).

### Sample Preparation

All stock solutions of BSA, TMAO, ThT, and ANS were prepared using the same potassium phosphate buffer (15 mM, pH 6.5). A stock solution of 20 mg/mL BSA was prepared by dissolving lyophilized BSA powder in buffer. The concentration was verified using a Thermo Scientific Nanodrop One Spectrophotometer (Wilmington, USA) along with the extinction coefficient (43,824 M^−1^ · cm^−1^) and molecular weight (66,430 g · mol^−1^) of BSA. The 20 mg/mL stock was diluted to 4 mg/mL and 0.4 mg/mL working solutions. Stock solutions of 400 mM TMAO, L-arginine, L-alanine and L-valine were prepared by reconstituting the dry powder in buffer. Stock solutions of the dyes ThT and ANS were prepared by reconstituting the dry powders in buffer to obtain final concentrations of 50 µM and 200 µM, respectively. The concentrations were verified using a Shimadzu UV-2600 spectrophotometer (Kyoto, Japan), a 50 µL Hellma fluorescence cuvette (Müllheim, Germany) and the extinction coefficient for ThT at 412 nm (36,000 M^−1^ · cm^−1^) and ANS at 350 nm (4,950 M^−1^ · cm^−1^). All stock solutions were stored at 4°C.

### Temperature Ramp with NanoDSF

Thermal stability profiles and apparent melting temperatures (T_m_) of Bovine Serum Albumin (BSA) were determined via nanoDSF using the NanoTemper Prometheus Panta instrument (Munich, Germany). Samples were loaded into Prometheus standard capillaries and heated from 25 °C to 90 °C at a constant ramp rate of 1°C/min. Formulations consisted of 1 mg/mL BSA prepared with 0, 50, 100, 150, and 200 mM of TMAO, L-arginine, L-alanine, or L-valine. The T_m_ values were calculated from the inflection points of the intrinsic fluorescence ratio (F_350_/F_330_) curves using the manufacturer's PR.Panta software.

### Fluorescence Spectroscopy

Both unfolding and aggregation mechanics were tracked with the external dyes ANS and ThT respectively. Fluorescence measurements were performed using a Fluorolog FL3-22 spectrofluorometer (New Jersey, USA) coupled with a Quantum Northwest TC 125 temperature-controller (Washington, USA). All samples were measured in a 50 μL Hellma fluorescence cuvette (Müllheim, Germany). Incubation of samples was conducted using a Bio-Rad Laboratories PTC Tempo 96 Thermal Cycler (California, USA). The lid temperature was set 5 °C above the block temperature to avoid evaporation of the samples.

### ANS and ThT Fluorescence

External dye fluorescence was carried out using 0.2 mg/mL of BSA for ANS and 2 mg/mL BSA for ThT.

Excipient screening contained samples with 200 mM of TMAO, L-arginine, L-alanine, or L-valine, which were incubated at 60 °C for ANS and 63 °C for ThT, for 0, 3, 5, 10, 15 and 25 min.

Arrhenius graph fitting was conducted using a temperature of 55, 58, 60, 63, 65 °C for ANS and 58, 59, 60, 63, 65, 70 °C for ThT at various time points.

Samples were prepared by transferring 90 μL of samples to 200 μL Eppendorf tubes and incubating at the respective temperatures. After incubation, samples were immediately placed on ice. Then 10 μL of 200 μM ANS, or 50 μM ThT were added to each sample, for a final concentration of 20 μM and 5 μM respectively. The samples were left for 30 min on the bench to allow dye binding, and the samples to equilibrate to the room temperature of 21°C. Fluorescence measurements were conducted using an excitation wavelength of 385 nm, emission wavelengths of 400 to 550 nm for ANS, and an excitation of 415 nm and emission of 425 to 550 nm for ThT. Emission spectra were collected with 1 nm bins, 1 nm excitation and emission slits for ANS, 4 nm for ThT, and an integration time of 0.15 s. The temperature of the cuvette holder was maintained at 21 °C throughout all measurements. All spectra were corrected for background fluorescence using the corresponding buffer controls.

### Arrhenius Analysis

The peak fluorescence intensities were found by fitting the emission spectrum with a gaussian curve in the Igor Pro 8 software (Oregon, USA):

2 $$y\left(x\right)={y}_{0}+A\cdot exp\left[\frac{-{\left(x-{x}_{0}\right)}^{2}}{2{\sigma }^{2}}\right]$$

where ${y}_{0}$ is the y offset, ${x}_{0}$ is the x offset, A is the scaling factor and ${\sigma }^{2}$ is the variance. The peak intensity ${y}_{peak}$ is given by:

3 $${y}_{peak}={y}_{0}+A$$

The peak fluorescence was plotted as a function of time, and the kinetics were obtained by fitting the data to a first order exponential decay function:

4 $${y}_{peak}\left(t\right)=c+A_0\cdot exp(-{k}_{obs}t)$$

where ${k}_{obs}$ is the unfolding rate, c is the y offset and $\mathrm{A}_{0}$ is the pre-exponential factor.

Activation energies were obtained by fitting the natural logarithm of the rate constants, $\mathrm{ln}\left({k}_{obs}\right)$, against reciprocal incubation temperature, *1/T*, in an Arrhenius plot:

5 $$\mathrm{ln}\left({k}_{obs}\right)=\mathrm{ln}\left({A}_{1}\right)-\frac{{E}_{a}}{RT}$$

where, *A*_*1*_ is the pre-exponential factor, ${E}_{a}$ is the activation energy, *R* is the universal gas constant, and *T* is temperature in Kelvin.

### Temperature Ramp with Fluorometer

Melting temperature studies were conducted in triplicates. For each measurement, an 80 µL aliquot of 0.2 mg/mL BSA was transferred to the fluorescence cuvette and then sealed with a lid to prevent evaporation. The sample was subjected to a temperature ramp, by piezoelectrically heating the cuvette chamber from 25 to 97 °C in increments of 3°C. At each temperature, the sample was allowed to equilibrate for 30 s before the emission spectrum was measured. Fluorescence measurements were conducted using an excitation wavelength of 295 nm, emission wavelengths ranging from 300 to 450 nm with 1 nm bins, 2 nm excitation and emission slits, and an integration time of 0.15 s. All spectra were corrected for background fluorescence by subtracting the corresponding buffer controls.

The peak intensity was obtained by fitting the emission spectrum with a gaussian curve (Eq. 2 and 3).

The CSM was obtained with each emission spectra using the equation:

6 $$CSM=\frac{\sum {\lambda}_{i}{I}_{i}}{\sum {I}_{i}}$$

where ${I}_{i}$ is the fluorescence intensity and ${\lambda}_{i}$ is the wavelength.

### SEC-MALS

SEC-MALS measurements were completed using an Agilent 1200 Liquid Chromatography System (Andover, Massachusetts) coupled to a Wyatt miniDAWN Multi-Angle Light Scattering module (California, USA). Separation of samples was carried out using a Tosoh SuperSW3000 column (Tokyo, Japan). Measurements were conducted using potassium phosphate buffer (150 mM, pH 6.5) as the mobile phase, with a flow rate of 0.2 mL/min and an injection volume of 10 µL, giving a final run time of 32 min.

Samples were prepared by transferring 40 µL aliquots of 2 mg/mL BSA to 200 µL Eppendorf tubes and incubating as per dye binding studies, at 60 °C (0–90 min, increments of 10 min), 65 °C (0–60 min, increments of 10 min), and 70 °C (0–30 min, increments of 5 min). After incubation, an additional 40 µL of potassium phosphate buffer (15 mM, pH 6.5) was added, yielding a final BSA concentration of 1 mg/mL. Measurements were conducted in triplicates. The Wyatt software program Astra 8.1.0 (California, USA) was used for data analysis. To determine the molecular weight of corresponding peaks in the chromatogram, the light scattering data was fit to the Zimm equation:

7 $$\frac{Kc}{R\left(\theta \right)}=\frac{1}{{M}_{w}\cdot P(\theta )}+2{A}_{2}c$$

where *K* is a system constant, *c* is concentration measured by the UV-detector at 280 nm, *R(θ)* is the excess Rayleigh ratio, *M*_*w*_ is the molecular weight, *A*_*2*_ is the second virial coefficient, and *P(θ)* is a theoretically derived form factor. Concentration was determined using the extinction coefficient for BSA of 0.6670 mL · mg^−1^ · cm^−1^. The molecular weight populations were determined using Igor Pro 8 by fitting the area under the UV curve for each peak.

### Fraction Isolation and Intrinsic Tryptophan Spectroscopy

Fractions of monomeric (19–20 min), dimer/trimer (17–18 min), and tetramer/pentamer (13–16 min) species were isolated using an automated fraction collector from a BSA sample incubated at 70 °C for 30 min. Intrinsic tryptophan fluorescence was measured using a Shimadzu RF-5301PC fluorometer (Kyoto, Japan), and a 50 μL Hellma fluorescence cuvette (Müllheim, Germany). The excitation wavelength was set to 295 nm, and the emission spectra were collected from 300–450 nm using 1 nm bins, and 3 nm excitation and emission slits. All spectra were corrected for background fluorescence by subtracting from a buffer sample.

### Unfolding Molecular Dynamics Simulations

High temperature unfolding molecular dynamics simulations were performed using GROMACS 2024.3 patched with PLUMED 2.11.0 [44, 45]. The structure of the BSA protein was prepared by extracting a single monomer from the crystal structure of the BSA dimer (PDB ID: 4F5S) [46]. The CHARMM36m force field and TIP3P water model were employed to model protein atoms and water molecules [47]. Protonation states were assigned based on PROPKA3.1 predictions at a pH of 7.2 [48]. The N- and C- termini were treated as charged, corresponding to NH_3_^+^ and COO^−^ respectively. All 17 disulfide bonds between cysteine residues were built.

Hydrogen mass repartitioning was applied to enable a 4 fs integration timestep [49]. The monomer was placed in a cubic periodic box with 1 nm spacing between the protein surface and the box edge. The system was solvated in 0.15 M NaCl to reflect physiological conditions. The final system contained 1 BSA, 147 Na^+^ ions, 131 Cl^−^ ions, and 42,733 water molecules.

The system was energy minimized using the steepest descent algorithm until the maximum force on any atom was less than 500 kJ/mol/nm. The particle mesh Ewald (PME) method was employed to manage long-range electrostatic interactions, with a real-space cutoff of 1.2 nm for both electrostatic and van der Waals interactions. The system was equilibrated in the NVT (Number of particles, Volume, Temperature) and NPT (Number of particles, Pressure, Temperature) for 200 ps and 400 ps respectively, using the leap-frog integrator, the V-rescale thermostat at 500 K and the C-rescale barostat at 1 bar. All heavy protein atoms were restrained with a harmonic potential of 1000 kJ/mol/nm^2^. At the end of equilibration, the final box edge length was approximately 11.1 nm.

Production simulations were conducted in the NPT ensemble using the same parameters without any positional restraints on the protein. A total of 4 independent simulations were run for 10 ns. Simulation frames were recorded at 100 ps intervals. Structures of the native as well as unfolded conformations were further analyzed with the Aggrescan3D webserver [24], as well as FreeSASA [25] to determine aggregation prone residues.

## Supplementary Information

Below is the link to the electronic supplementary material.

ESM 1 (PDF 1.69 MB)

## Data Availability

Simulation and structure files can be found at https://github.com/samueltien/BSAunf. All other data will be made available at request.
