# Supplementary material for: Polar vs Non-polar Excipients in BSA Aggregation
Source: Pharm Res. 2026 Oct 7;43(10):3879–93. doi: 10.1007/s11095-026-04204-2 (PMC13736487; doi:10.1007/s11095-026-04204-2)
Supplement: Supplementary file 1 [file 11095_2026_4204_MOESM1_ESM.pdf]

Supporting Information for Publication

Polar vs Non-polar Excipients in BSA Aggregation

Samuel Tien[1], Simone Nørgaard Krabbe[1*], Annika Sollann Sørensen[1*], Thomas Balle[1], Veysel Kayser[1]

[1]The University of Sydney, Faculty of Medicine and Health, Sydney School of Pharmacy, Sydney, NSW 2006, Australia

[*]Current address: The University of Copenhagen, Faculty of Health and Medical Sciences, Department of Pharmacy, 2100 Copenhagen, Denmark

Corresponding author: veysel.kayser@sydney.edu.au

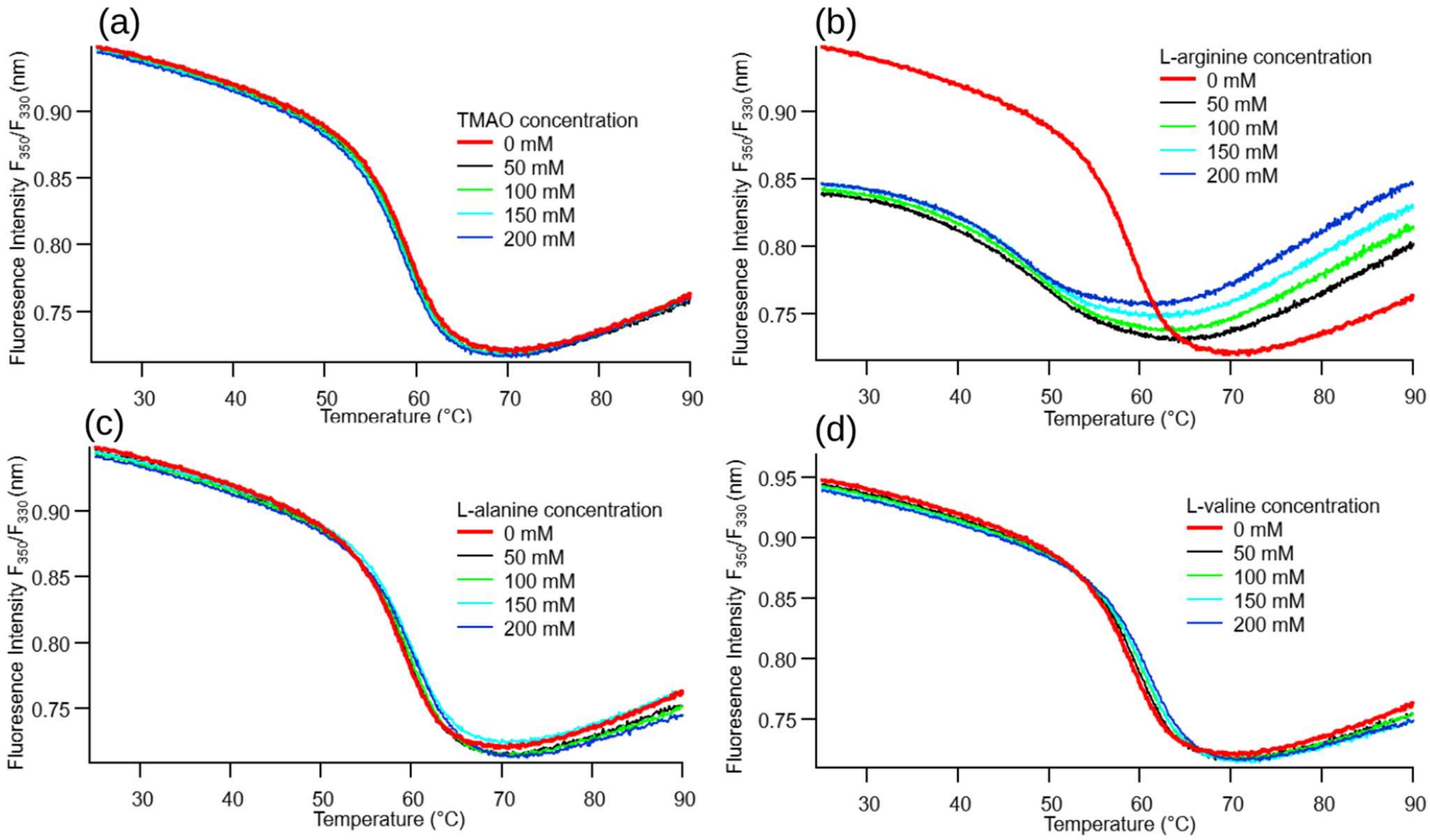

Supplementary Fig. 1: Full nanoDSF thermal degradation profiles of BSA across varying excipient concentrations. Temperature ramps were performed with 0, 50, 100, 150, and 200 mM of **(a)** TMAO, **(b)** L-arginine, **(c)** L-alanine, and **(d)** L-valine.

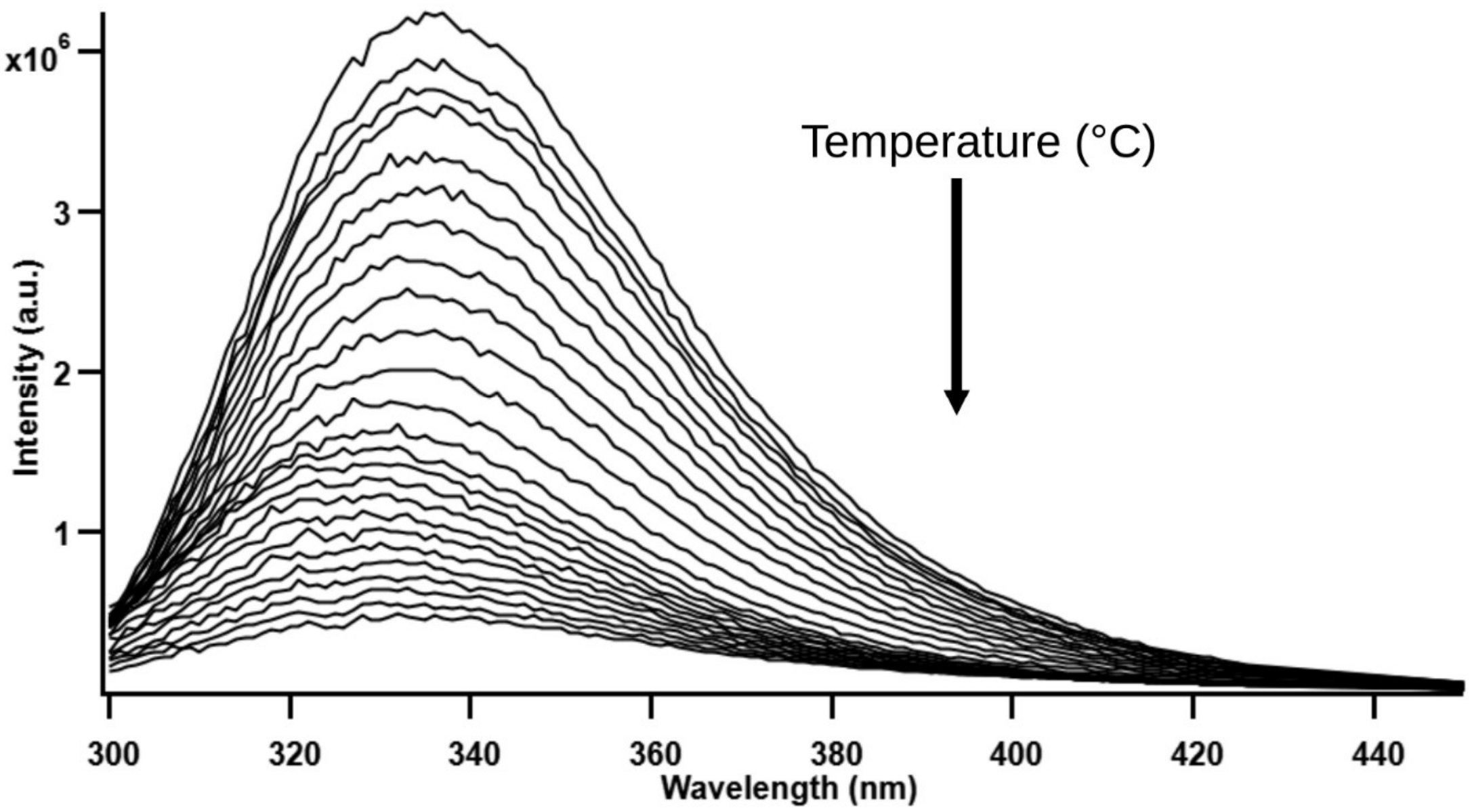

Supplementary Fig. 2: BSA intrinsic tryptophan fluorescence emission spectra of BSA collected from a temperature ramp of 25-97 °C in 3 °C intervals for 1 representative sample.

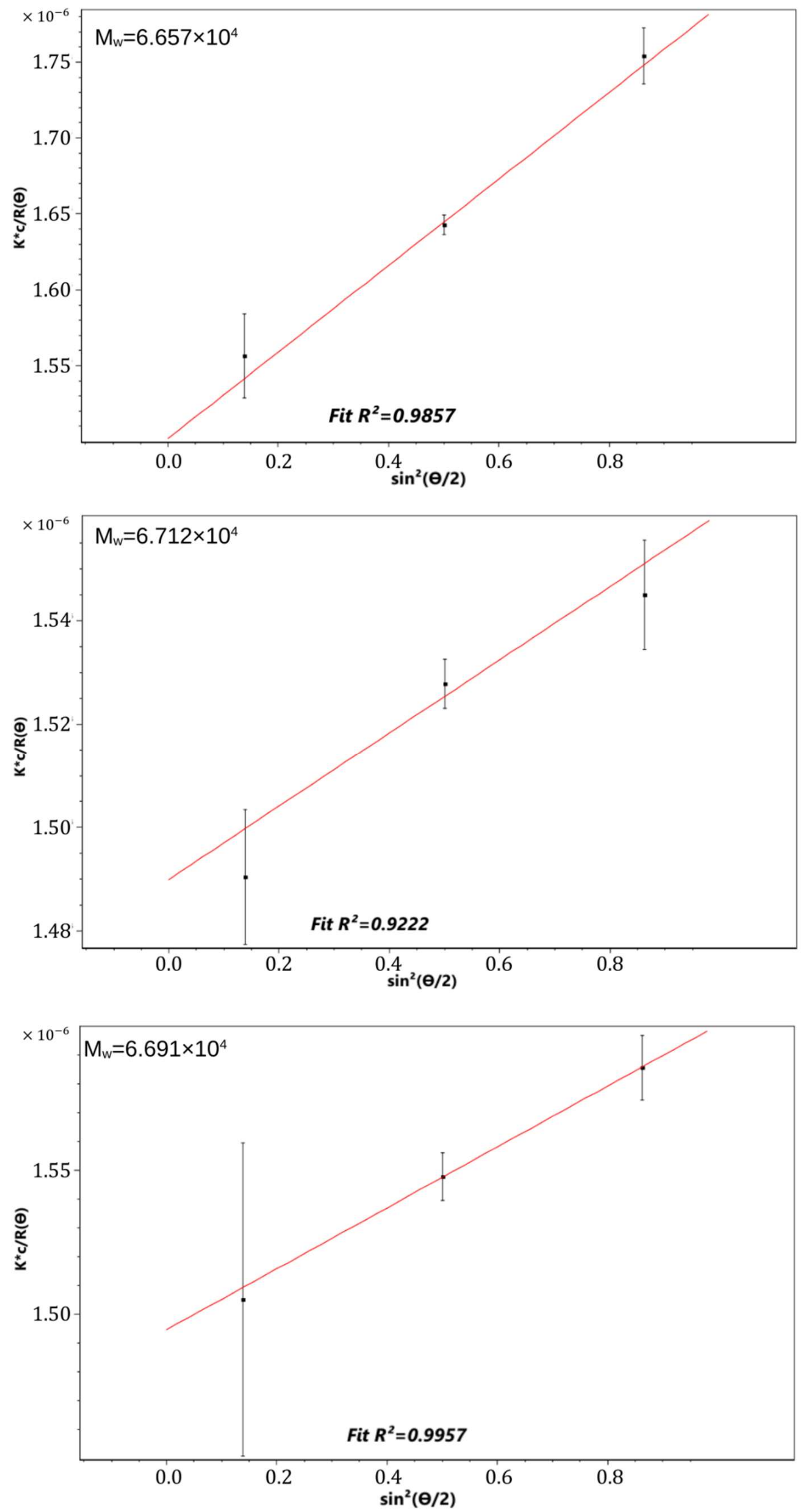

Supplementary Fig. 3: Zimm plot analysis for the BSA monomer. Calculated molecular weights are highly reproduceable (6.69±0.03 · 10^4 g/mol, RSD <1.5%)

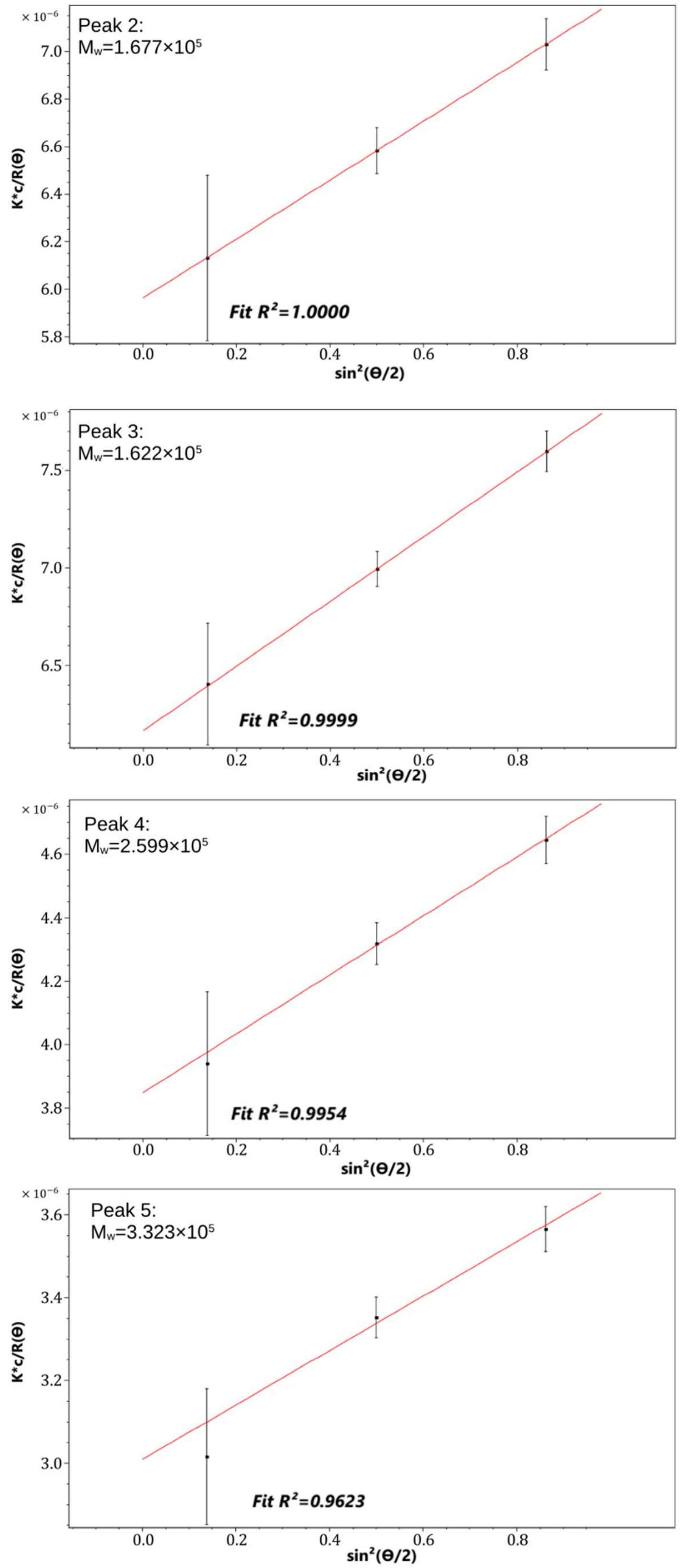

Supplementary Fig. 4: Zimm plot analysis of each aggregated species, showing the calculated molecular weights.

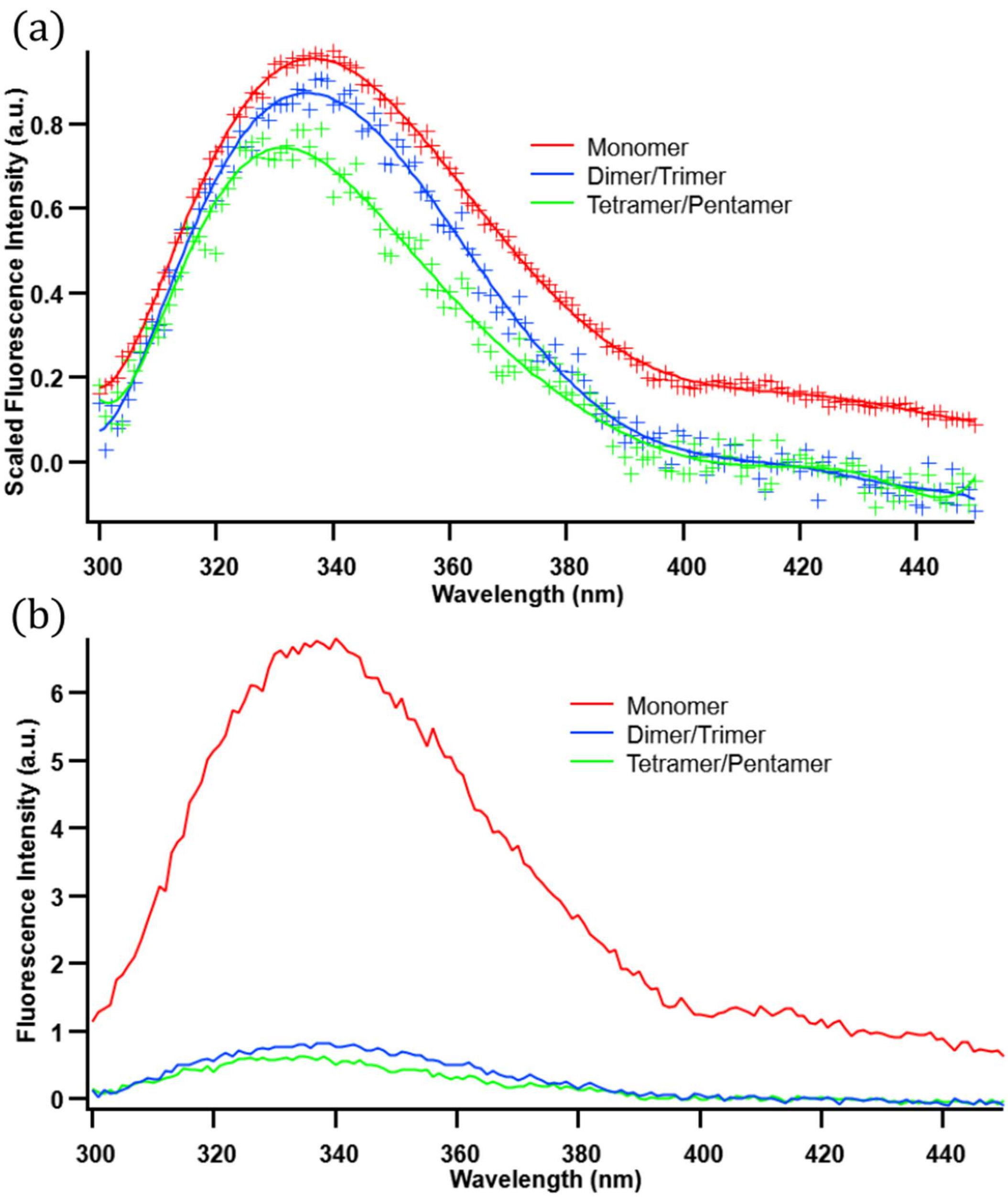

Supplementary Fig. 5: Background subtracted intrinsic tryptophan fluorescence emission spectra of each isolated BSA oligomeric species. **(a)** Fluorescence spectra with scaled intensities for comparison of peak wavelengths. Solid lines represent polynomial fits to the experimental data. **(b)** Raw unscaled fluorescence spectra.

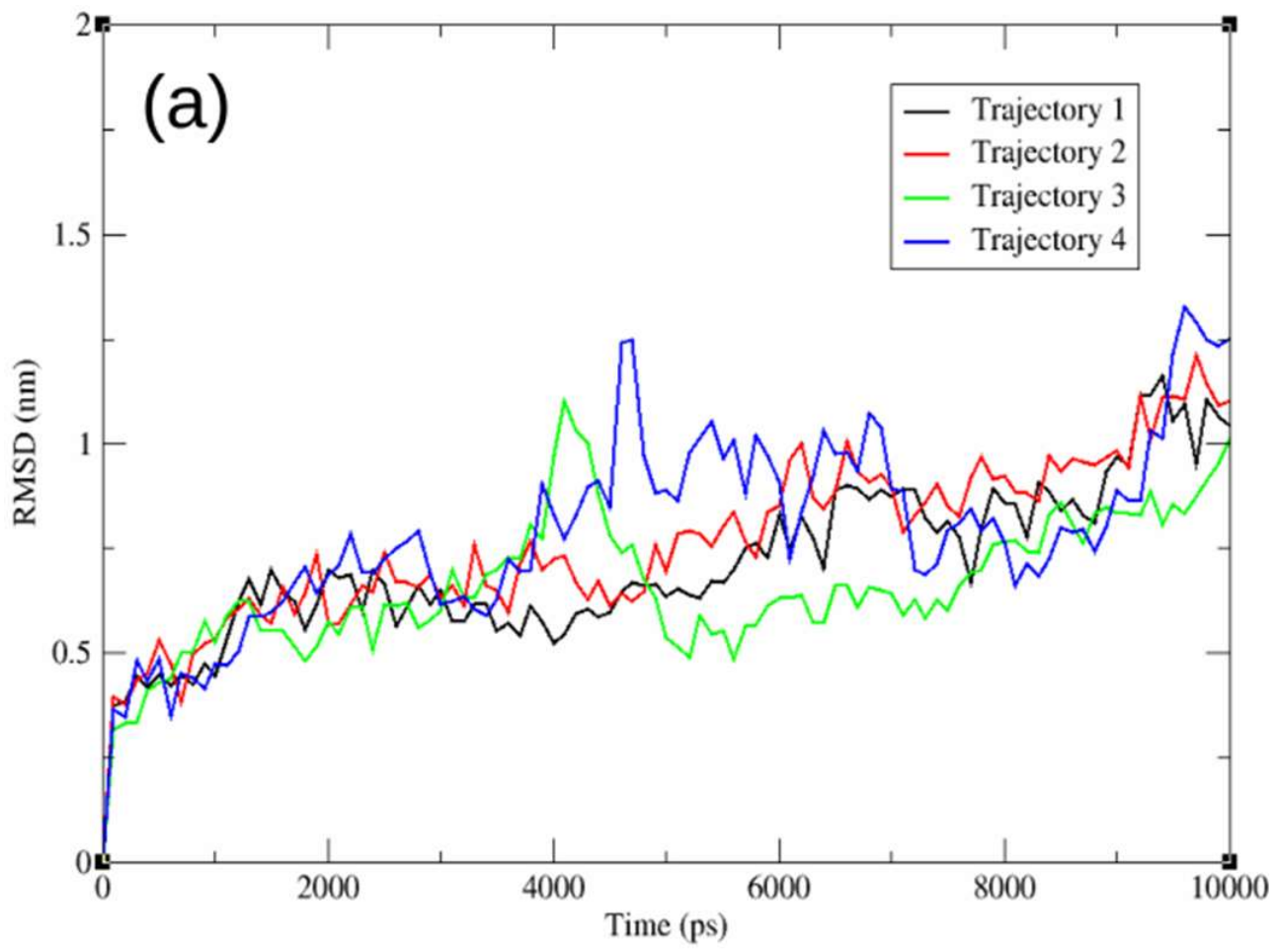

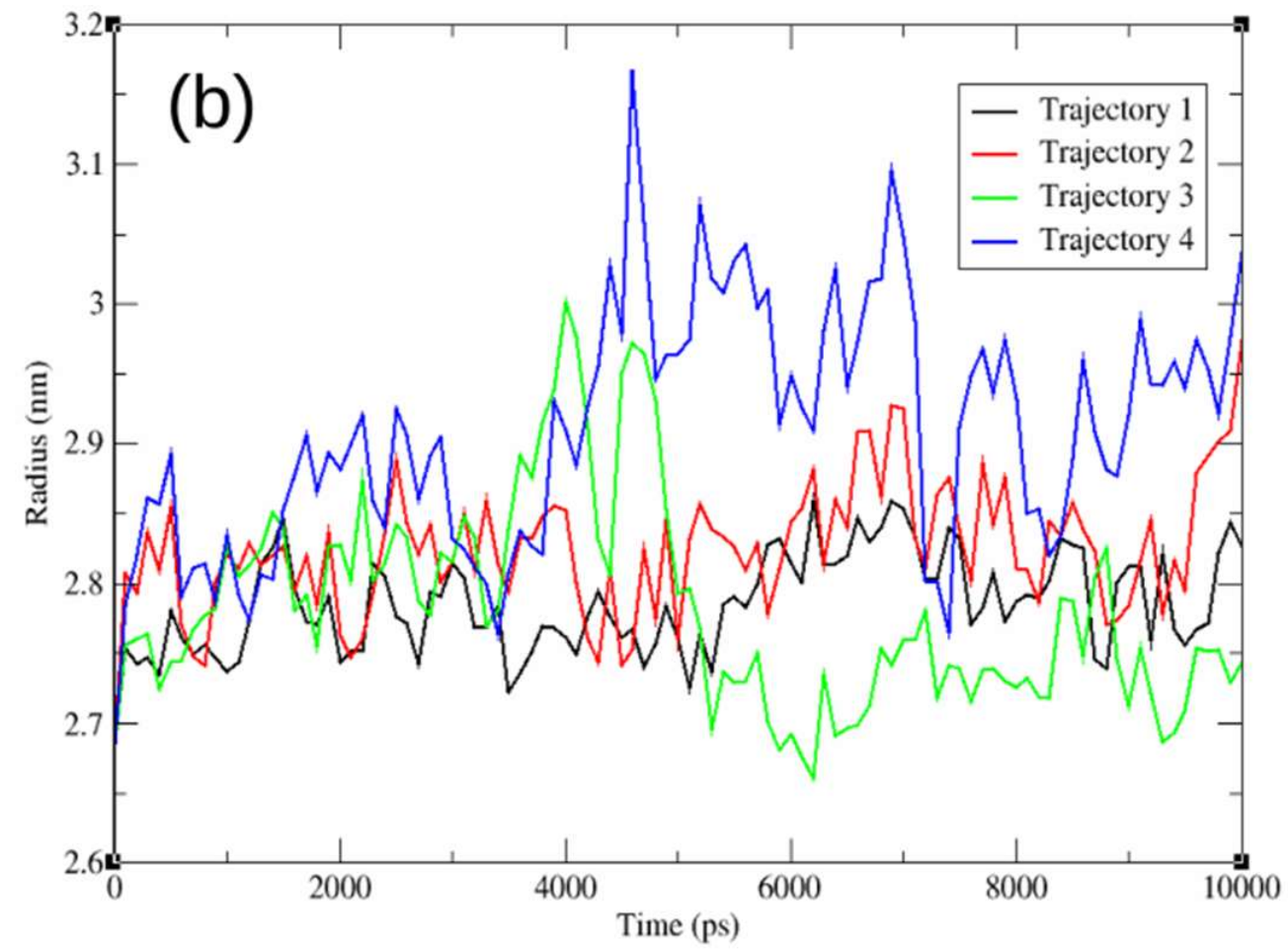

Supplementary Fig. 6: Trajectory analysis of unfolding simulations showing the change of **(a)** RMSD and **(b)** Rg over time.

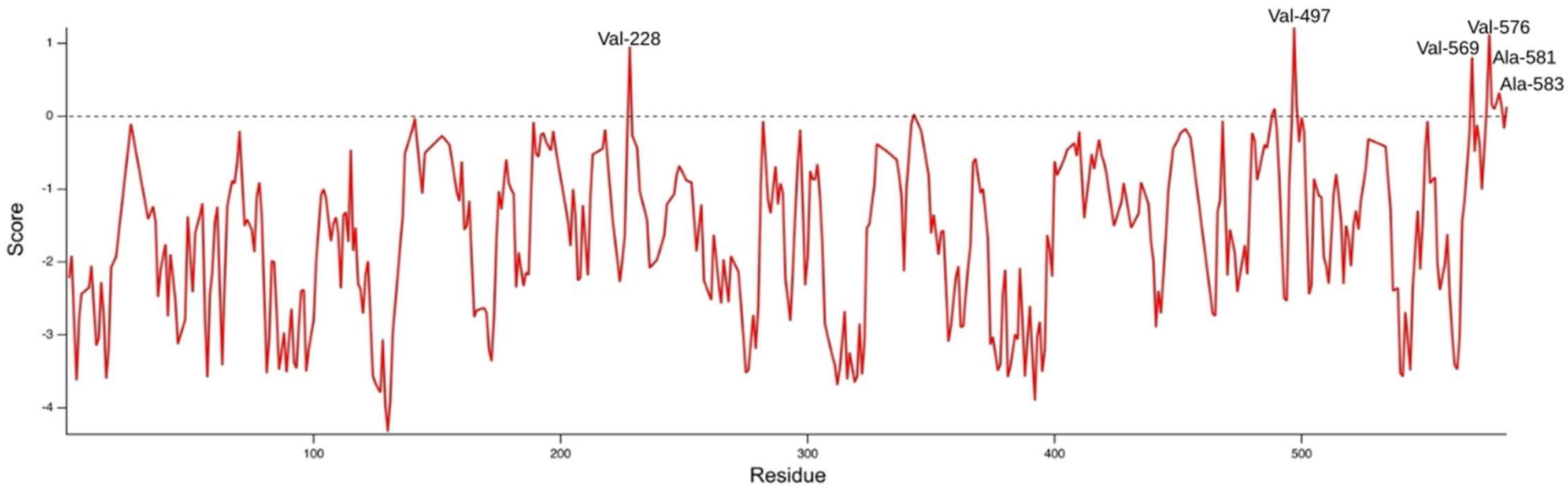

Supplementary Fig. 7: Aggrescan3D profile of the native BSA structure. Individual residue-level Aggrescan3D scores are plotted, with specific hot spots labelled where values are positive.

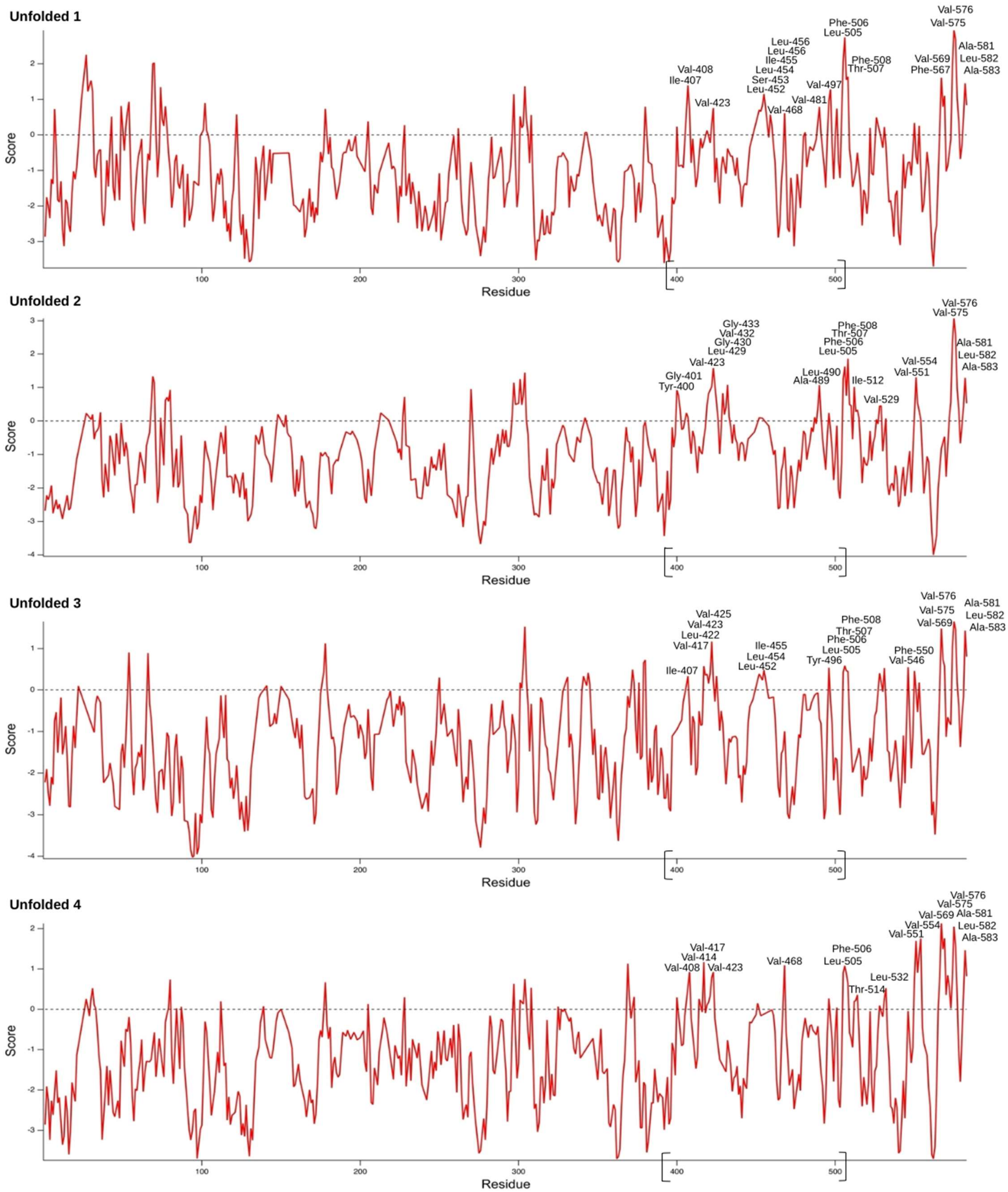

Supplementary Fig. 8: Aggrescan3D profile of the unfolded structures. Individual residue-level Aggrescan3D scores are plotted, with specific hot spots labelled in both the C-terminus (491-583) and the 384-490 residue index region where values are positive.

Supplementary Table I: Summed Aggrescan3D and FreeSASA scores for native and unfolded structures.

Structure	Total Aggrescan3D score	Total SASA (Å^2)	Non-polar SASA (Å^2)	Polar SASA (Å^2)
Native	-693.5	29259.0	15872.1	13222.1
Unfolded 1	-518.0	37195.7	21445.6	15391.4
Unfolded 2	-505.1	32704.9	18902.0	13486.9
Unfolded 3	-594.3	33587.6	19107.1	14272.1
Unfolded 4	-588.0	35665.8	20350.3	15046.7
